# Emergence of the third-generation cephalosporin-resistant hypervirulent *Klebsiella pneumoniae* due to the acquisition of a self-transferable *bla*_DHA-1_-carrying plasmid by an ST23 strain

**DOI:** 10.1080/21505594.2018.1456229

**Published:** 2018-04-23

**Authors:** Yingzhou Xie, Lijun Tian, Gang Li, Hongping Qu, Jingyong Sun, Wei Liang, Xiaobin Li, Xiaoli Wang, Zixin Deng, Jialin Liu, Hong-Yu Ou

**Affiliations:** a State Key Laboratory of Microbial Metabolism, School of Life Sciences & Biotechnology, Shanghai Jiao Tong University, Shanghai, China; b Department of Critical Care Medicine, Ruijin Hospital, Shanghai Jiao Tong University School of Medicine, Shanghai, China; c Department of Laboratory Medicine, Jinshan Hospital Fudan University, Shanghai, China; d Department of Clinical Microbiology, Ruijin Hospital, Shanghai Jiao Tong University School of Medicine, Shanghai, China; e Department of Laboratory Medicine, the Second People's Hospital of Lianyungang, Jiangsu, China; f Shanghai Key Laboratory of Veterinary Biotechnology, School of Life Sciences & Biotechnology, Shanghai Jiao Tong University, Shanghai, China

**Keywords:** *bla*_DHA-1_, conjugation transfer, comparative genomic analysis, hypervirulent *Klebsiella pneumoniae*, multidrug resistant plasmid

The bacterial species *Klebsiella pneumoniae* is an increasingly important human pathogen. Dramatic increases in the levels of multidrug resistance (MDR) associated with this species [1], particularly resistance to carbapenems and third-generation cephalosporins, pose an emerging global problem [2]. The common drug-resistant strains of *Klebsiella pneumoniae* are ST11 or ST258, which were identified by multilocus sequence typing (MLST) [3,4]. Notably, hypervirulent variants of *K. pneumoniae* (hvKP), which cause severe infections such as liver abscesses, endophthalmitis, meningitis, osteomyelitis, necrotizing fasciitis in healthy and ambulatory individuals [5], have been increasingly reported recently. hvKP often exhibit hypermucoviscosity and mainly belong to ST23 (by MLST) for the K1 capsular serotype and to ST86 and ST375 for the K2 serotype [5,6]. In contrast to classic *K. pneumoniae* (cKP), hvKP usually show a high level of susceptibility to the antimicrobial agents used for clinical treatment, except for ampicillin [7]. However, in the last decade, hvKP with high degrees of drug resistance have increasingly emerged, even in the well-documented K1 and K2 serotype hvKP strains [8–12]. So far, there are two papers reporting hypervirulent *K. pneumoniae* that produced DHA-1 beta-lactamase and ESBL in France [8] and Korea [12], respectively; however, the genetics investigationor genome backgrounds remains to be characterized. Recently the classic ST11 carbapenem-resistant *K. pneumoniae* strain has been reported to have acquired a virulence plasmid, resulting in a carbapenem-resistant hypervirulent variant of ST11 (hv-MDR-KP) [13].

In cKP, the acquired resistance genes are found mainly embedded on plasmids, for example, the *bla*_KPC-2_ gene that confers carbapenems resistance [14] and the *mcr-1* gene that confers colistin resistance [15]. Similarly, plasmids are also the major carriers for emerging drug-resistance genes in multidrug resistant hvKP (MDR hvKP). The *bla*_CTX-M-3_ gene was found on an IncL/M plasmid, in an ST86 and K2 hvKP isolated in France [8]. A carbapenem-resistant ST23 hvKP with serotype K1 was reported in China, and its *bla*_KPC-2_ gene was located on a non-transferable plasmid [10]. Plasmids carrying resistance genes with transferability to hvKP were also observed. The conjugation between typical carbapenemase-producing cKP (ST258) and K2 hvKP (ST65) showed that the plasmid carrying the *bla*_KPC-2_ or *bla*_KPC-3_ genes could be transferred to and retained in hvKP [16]. Siu *et al.* reported a case of recurrent liver abscess caused by hvKP and successfully mimicked the *bla*_CMY-2_ gene transferred from *E. coli* to K2 hvKP *in vitro* [17]. However, so far, there have been no reports of hvKP-borne resistance plasmids that are transferable to other hvKP strains.

Here, we present hypervirulent *K. pneumoniae* clinical strain RJA166 with resistance to all the third-generation cephalosporins and to the combination of β-lactam and β-lactamase inhibitors. RJA166 is a typical hvKP with a K1 capsular serotype and ST23 sequence type. The *bla*_DHA-1_ gene, as well as three other AMR genes, were identified as being on the natural plasmid pRJA166a and this resistance plasmid was subsequently determined to be transferable to the K1 and K2 hvKP strains and to the ST11 cKP.

First, we investigated the virulence of *K. pneumoniae* RJA166 in the *G. mellonella* larvae and mouse infection models. *K. pneumoniae* RJA166 was isolated from sputum specimen from a patient in Ruijin Hospital, Shanghai, China. It showed a viscous string longer than 40 mm (Figure S1a) by the string test. It was also determined to be ST23 and a capsular serotype K1 strain using PCR in our retrospective experiment. RJA166 was then determined to be as virulent as *K. pneumoniae* NTUH-K2044 in the killing assay on *Galleria mellonella* larvae, with the cKP HS11286 (ST11 and KL103 serotype [18]) as control (Figure S1b, Table S1). For further confirmation of RJA166 hypervirulence, a mouse lethality assay was performed. RJA166 showed an LD50 of 2.3 × 10^2^ CFU in BALB/c mice, which was comparable to hypervirulent NTUH-K2044 strain [19,20]. The data from the killing assays on both mice and *G. mellonella* show that RJA166 had the same virulence level as NTUH-K2044, which has been shown to be highly pathogenic in a previous study [21], more so than the classic ST11 *K. pneumoniae* [14]. In addition, the liver, spleen, and kidney of the mouse which was challenged by 10^3^ CFU *K. pneumoniae* RJA166 and sacrificed 3 days after infection, were taken for the further histopathological examination. Compare to the control, the infected liver showed multiple microabscesses, with infiltration of inflammatory cells, including neutrophils and monocytes mainly in the part of the portal center infiltration mode, accompanied with vacuolization, focal hyperemia, and hemorrhage. Eosinophilic liver abscess exhibited pale stained nucleus, irregular shape, with the parenchyma becoming looser due to infiltration of inflammatory cells, whilst the normal liver cells have more obvious boundaries (Figure S2a and S2d). In comparison with the control kidney, there was local renal capsule inflammatory cell infiltration, glomerular lesions in varying degrees of atrophy, mild swelling of renal tubular epithelial cells, mild expansion of the lumen observed in the infected group (Figure S2b and S2e). The infected spleen showed white pulp atrophy, irregular structure, reduced number of lymphoid follicles. The remaining follicular morphology was also less regular, with large peripheral macrophage accumulation. Mild red pulp hyperemia, with slightly scattered thrombosis, were also observed (Figure S2c and S2f).

Secondly, we wanted to address the resistance of hvKP RJA166 to third-generation cephalosporins. The MIC data generated by the VITEK 2 Compact system showed that RJA166 was resistant to first-, second- and third-generation cephalosporins; aztreonam; and a combination of β-lactam and β-lactamase inhibitors (Table 1), suggesting that RJA166 is a ST23 MDR-hvKP. RJA166 also displayed low-level resistance to ertapenem but was susceptible to imipenem (Table 1). Additionally, this strain was determined to be susceptible to aminoglycosides and fluoroquinolones, such as amikacin, gentamycin, tobramycin and ciprofloxacin (Table 1). The resistance profile of RJA166 suggested the potential existence of a gene encoding the AmpC β-lactamase in its genome.

**Table 1.** Antimicrobial susceptibility test of the hypervirulent *K. pneumoniae* RJA166, RJF293 and pRJA166a/RJF293H transconjugants.^*^

	MIC (μg/ml) / Antimicrobial Susceptibility^†^					
Antimicrobial Agents	RJA166	RJF293	RJF293H	Tc1^‡^	Tc2^‡^	Tc3^‡^
*Penicillin*						
Ampicillin	≥32 / R	≥32 / R	16 / R	≥32 / R	≥32 / R	≥32 / R
*β-lactam/β-lactamase inhibitor combinations*						
Ampicillin/Sulbactam	≥32 / R	8 / S	4 / S	≥32 / R	≥32 / R	≥32 / R
Piperacillin/Tazobactam	8 / R	≤4 / S	≤4 / S	≤4 / S	≤4 / S	≤4 / S
*Cephems*						
Cefazolin	≥64 / R	≤4 / S	≤4 / S	≥64 / R	≥64 / R	≥64 / R
Cefotetan	≥64 / R	≤4 / S	≤4 / S	8 / S	16 / S	16 / S
Ceftazidime	≥64 / R	≤1 / S	≤1 / S	≥64 / R	≥64 / R	≥64 / R
Ceftriaxone	≥64 / R	≤1 / S	≤1 / S	8 / R	8 / R	8 / R
Cefepime	2 / S	≤1 / S	≤1 / S	≤1 / S	≤1 / S	≤1 / S
*Monobactams*						
Aztreonam	≥64 / R	≤1 / S	≤1 / S	≥64 / R	≥64 / R	≥64 / R
*Carbapenems*						
Ertapenem	2 / R	≤0.5 / S	≤0.5 / S	≤0.5 / S	≤0.5 / S	≤0.5 / S
Imipenem	≤1 / S	≤1 / S	≤1 / S	≤1 / S	≤1 / S	≤1 / S
*Aminoglycosides*						
Amikacin	≤2 / S	≤2 / S	≤2 / S	≤2 / S	≤2 / S	≤2 / S
Gentamycin	≤1 / S	≤1 / S	≤1 / S	≤1 / S	≤1 / S	≤1 / S
Tobramycin	≤1 / S	≤1 / S	≤1 / S	≤1 / S	≤1 / S	≤1 / S
*Fluoroquinolones*						
Ciprofloxacin	0.5 / S	≤0.25 / S	≤0.25 / S	0.5 / S	0.5 / S	0.5 / S
Levofloxacin	1 / S	≤0.25 / S	≤0.25 / S	1 / S	1 / S	1 / S
*Nitrofurans*						
Nitrofurantoin	128 / R	128 / R	128 / R	256 / R	256 / R	256 / R
*Folate pathway inhibitors*						
Trimethoprim/Sulfamethoxazole	≤20 / S	≤20 / S	≤20 / S	≤20 / S	≤20 / S	≤20 / S

* *K. pneumoniae* pRJA166a/RJF293H transconjugants (Tc1-3) harbored a natural 231-kb plasmid carrying *bla*_DHA-1_ (pRJA166a). The minimal inhibitory concentration was determined by the VITEK2 compact system.

† Bacterial antimicrobial susceptibility was interpreted as per the CLSI guidelines 2016 (M100-S26). R: resistant; S: susceptible.

‡ Tc1-3 denote the three pRJA166a/RJF293H transconjugants randomly selected from conjugation assay between *K. pneumoniae* RJA166 and RJF293H.

The resistance of hvKP RJA166 in a murine model was also determined by an antibiotic treatment assay. Ceftazidime (40 mg/kg) and imipenem (20 mg/kg) were administered to BALB/c mice intraperitoneally challenged by *K. pneumoniae* RJA166. The infected mice treated with ceftazidime displayed higher mortality (70%) than the mice in the imipenem group (30%). No survival was observed in the group treated with 0.9% saline (Fig. 1a) as a control. The bacterial loads analysis in murine tissues was determined to be consistent with the survival analysis, by quantitative real-time PCR with primers of bacterial *gyrB* gene and the murine β-actin gene (Table S2). The mice treated with ceftazidime showed higher bacterial loads in kidneys (*p* = 0.0383, by Student's t test), spleen (*p* < 0.0001) and liver (*p*<0.0001) (Fig. 1b) than the mice in the imipenem group. This demonstrates that the drug resistance of the ST23 MDR-hvKP RJA166 contributes to bacterial survival in the murine model under the treatment of third generation cephalosporins.

**Figure 1.**
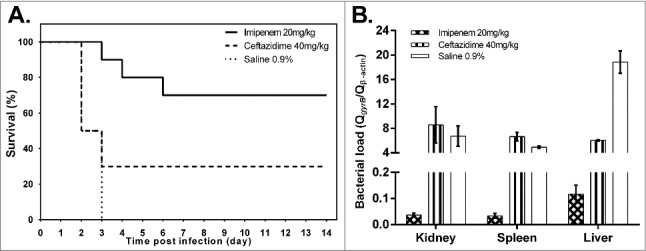
Effect of ceftazidime and imipenem on *K. pneumoniae* RJA166 infection in murine model. (a) The survival curve of the BALB/c mice infected with 1.8 × 10^3^ CFU of *Klebsiella pneumoniae* RJA166 with different treatments. In the 14 days after bacteria challenge, mice treated by imipenem (20 mg/kg), ceftazidime (40 mg/kg) and saline (0.9%) showed mortalities of 30%, 70% and 100%, respectively. (b) The bacterial load of murine tissues in the mice treated with different antibiotic. The results were calculated by dividing the quantity of bacterial gyrase gene by the quantity of murine β-actin gene. The bacterial loads in the kidneys, spleens and livers from the mice treated with ceftazidime were significantly higher than those in the mice treated with imipenem, with the *p* value 0.0383, <0.0001 and <0.0001, respectively, as generated by Student's t test.

Thirdly, our genome analysis showed that the hvKP RJA166 carries a conjugative resistance plasmid pRJA166a. The whole genome sequencing showed that the ST23 MDR-hvKP RJA166 genome consists of a circular chromosome and three circular plasmids (Table S3; supplementary Materials and Methods), including the antibiotic resistance plasmid pRJA166a (230 Kb in size; Fig. 2), the virulence plasmid pRJA166b (229 Kb; Figure S3a) and the plasmid pRJA166c with unknown function (111 Kb; Figure S3b and S3c). The RJA166 chromosome exhibits similar genetic background to the other sequenced K1 hvKP strains, as determined by the core chromosomal SNP-based phylogenetic analysis of 107 completely sequenced *K. pneumoniae* strains (Figure S4). The resistance plasmid pRJA166a exhibited a genetic background distinct from to the virulence plasmids frequently found in the sequenced K2 or K1 serotype hvKP strains. We thus presumed that the acquisition of a *bla*_DHA-1_-carrying plasmid by K1 hvKP resulted in a multidrug-resistant hypervirulent variant.

**Figure 2.**
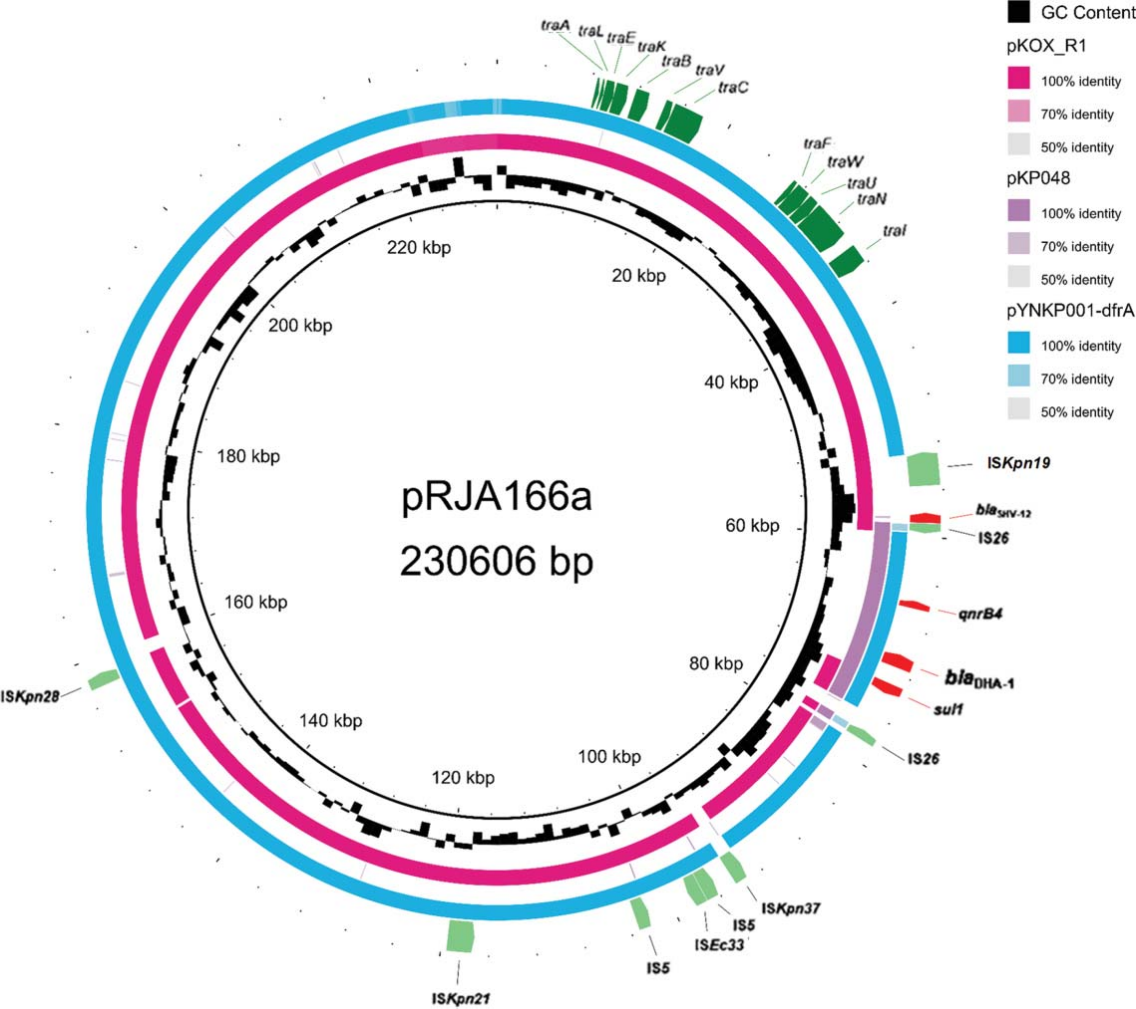
The genetic context of the *bla*_DHA-1_ gene in the resistance plasmid pRJA166a of *K. pneumoniae* RJA166. The plasmid shares the backbone of two previously reported plasmids pYNKP001-dfrA of *Raoultella ornithinolytica* YNKP001[ref. 22] and pKOX_R1 of *Klebsiella michiganensis* E718 [ref. 23]. The predicted antibiotic resistance gene is shown in red, the tra gene cluster is in dark green, and the IS element is shown in light green.

We then typed the incompatible group of pRJA166a as IncHI5 and found that its backbone was highly homologous to the previously reported IncHI5-group plasmids, namely, pYNKP001-dfrA of *Raoultella ornithinolytica* YNKP001 [ref. 22] and pKOX_R1 of *K. michiganensis* E718 [ref. 23]. Notably, in addition to the region homologous to pKOX_R1 and pYNKP001-dfrA (Figure S5), pRJA166a has a 26-kb accessory region (*RJA_27650..RJA_27825*) associated with antibiotic resistance genes, integrons, transposons and insertion sequence-based mobile elements (Fig. 3). Across this mosaic-like, three-module region, the *bla*_DHA-1_ gene is present on Module II of the accessory resistance region, a relatively large portion of this 26-kb region. The AmpC-type β-lactamase gene *bla*_DHA-1_, first reported in 1997 [ref. 24], confers resistance to third-generation cephalosporins on the host. This gene is frequently carried by plasmids belonging to the IncH and IncR incompatibility groups. The genetic context of *bla*_DHA-1_ is organized as a conserved structure: *sul1-qacEΔ1-ampR-bla*_DHA-1_-*pspABCDF-qnrB4.* This structure is often associated with the class I integron and IS*CR1*; however, a complete integron was not found; there was no 5′-CS or *int* gene present in pRJA166a. This gene is highly conserved in the *bla*_DHA-1_-carrying plasmids of *Klebsiella* strains [25,26]. Module II is located upstream of IS*CR1* in pRJA166a and in the IncF plasmid pKP048 [ref. 27], but downstream of IS*CR1* in pYNKP001-dfrA. Additionally, the IS*26* elements bracketed Module II-III are in opposite orientation, but no target site duplication was observed. Due to their opposite orientations, we propose that these IS*26* elements and the Module II-III sandwiched between them might constitute a new composite transposon capable of being disseminated to other replicons [28]. Thus, given the highly mosaic-like structure of the pRJA166a accessory region, we suggest that the present entity represents a relic of multiple past insertions, deletions and recombination events that may have since acquired multidrug resistance genes.

**Figure 3.**
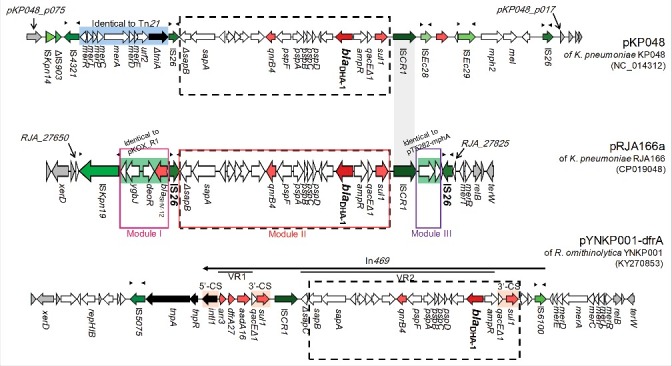
Organizational map of the 26-kb accessory resistance region (*RJA_27650…RJA_27825*) on pRJA166a. Two other *bla*_DHA-1_-carrying plasmids with the same incompatibility group (IncHI5), pKP048 and pYNKP001-dfrA, were compared. The three modules (I–III) referred to in the text are shown as red boxes while the module that contains the conserved *bla*_DHA-1_ context is highlighted in the black boxes with dashed borders. The protein-coding regions are shown as arrows with different colours, where green indicates an IS element, black indicates integrase gene, red indicates antibiotic resistance gene and grey indicates flanking gene. Genes identical to the 3′-CS of class 1 integron are shown with an orange background. Inverted repeats of IS elements are represented as small black triangle above the arrow. VR1 and VR2 are the variable regions of In*469*, as defined by Liang *et al* [22].

To date, third-generation cephalosporins have been the main antibiotics for the treatment for of infections caused by hypervirulent *K. pneumoniae* [29]. Recently, the emergence of multidrug-resistant hypervirulent *K. pneumoniae* strains has been reported in different countries, which include a broad range of sequence types [8,11,30]. The ST23 MDR-hvKP strain RJA166 presented here is the first hvKP strain with resistances to the third-generation cephalosporins collected in the ICU of our hospital. This type of *K. pneumoniae* strain has been reported in Korea [12], but neither conjugation assay nor genetic background parsing was included in the previous publication. Thus, the approach of the emergence of multidrug resistant hypervirulent *K. pneumoniae* strains is still unclear. Our WGS data suggested that RJA166 carries three natural plasmids, including a resistance plasmid pRJA166a and a virulence plasmid pRJA166b. *K. pneumoniae* variants with both multidrug resistance and hypervirulence phenotypes potentially developed via two distinct approaches: (I) the multidrug-resistant hvKP (MDR-hvKP), origin from the hvKP; and (II) the hypervirulent multidrug-resistant *K. pneumoniae* (hv-MDR-KP), derived from the multidrug-resistant cKP. The development of the virulence and resistance traits of the ST23 MDR-hvKP presented here (approach I above) is distinct from that of the newly reported ST11 hv-MDR-KP [13] (approach II above).

Finally, we investigated the conjugative transfer of the *bla*_DHA-1_-containing plasmid pRJA166a between diverse *K. pneumoniae* strains. By filter mating, the ceftazidime resistance was found to be successfully transferred from the ST23 MDR-hvKP RJA166 to RJF293H, which is a hygromycin-resistant mutant from the ST374 and K2 serotype hvKP clinical strain RJF293 [ref. 7]. The MIC tests of three randomly selected *K. pneumoniae* pRJA166a/RJF293H transconjugants (Tc1-3) revealed that the resistance to cephalosporin antibiotics was elevated (Table 1). PCR amplifications of fragments from the *bla*_DHA-1_, *repA* and *traD* genes of pRJA166a confirmed that the antibiotic resistance dissemination transfer was mediated by pRJA166a (Figure S6c). Then, the *Xba*I-PFGE and S1-nuclease PFGE suggested that the increase in antimicrobial resistance of these transconjugants was due to the acquisition of the plasmid pRJA166a (Figure S6a-b). Finally, the transconjugation frequency of pRJA166a from RJA166 to RJF293H was determined to be approximately 2.6 × 10^−6^ transconjugants per donor cell. We subsequently assessed the stability of pRJA166a in *K. pneumoniae* RJA166 and RJF293H transconjugants by a continuous passage. After 375 generations (15 days) on the LB agar without antibiotic selection, no loss of the plasmids in either host was observed. We further performed a conjugation assay to explore whether pRJA166a was able to disseminate across an expanded collection of *K. pneumoniae*. Similarly, the expected transfer of pRJA166a from RJA166 (ST23 and K1) to its closely related recipient NTUH-K2044IT (a hygromycin-resistant derivative of ST23 and serotype K1 *K. pneumoniae* NTUH-K2044; Table S1) was observed with a frequency of approximately 5.5 × 10^−6^ transconjugants per donor. Remarkably, pRJA166a can also be transferred into the carbapenem-resistant cKP strain HS11286 (ST11 and KL103), by using its hygromycin resistant derivative HS11286YZ6 (Table S1). The transfer frequencies to HS11286YZ6 were determined to be approximately 2.7 × 10^−6^ transconjugants per donor. However, the conjugative transfer of pRJA166a from RJA166 to the frequently used *E. coli* recipients either HB101 or J53 was not detectable using the same conditions described above.

The transfer of resistance plasmids has been previously reported to occur either from hvKP to *E. coli* [10] or *vice versa* [16,17]. Our study showed the conjugative transfer of a resistance plasmid pRJA166a, which coded for the entire conjugation machinery, between hvKP. The entire plasmid pRJA166a transferred to both the K1 hvKP strain and the K2 hvKP strain, resulting in MDR-hvKP. Notably, the resistance plasmid pRJA166a kept its stability across the transconjugants. Although the pRJA166a acquisition comes at a fitness cost to the recipient (Figure S7a), the *G. mellonella* larvae infection assay confirmed that these obtained transconjugants are hypervirulent like the recipient (Figure S7b and S7c). Remarkably, pRJA166a was also transferable to the carbapenem-resistant ST11 cKP strain HS11286, which had already carried three resistance plasmids. These results indicated that the resistance plasmid pRJA166a carrying the *bla*_DHA-1_ gene could be disseminated among *K. pneumoniae*.

In conclusion, we investigated the genetic background and microbiological features of a hypervirulent clinical strain (RJA166) of *K. pneumoniae* with resistances to the third-generation cephalosporins (ST23 MDR-hvKP). The *bla*_DHA-1_-carrying natural plasmid from this K1 and ST23 hvKP that is transferable to hvKP and cKP might contribute to the rapid spread of drug resistance among *K. pneumoniae* and other bacterial pathogens.

## Nucleotide sequence accession numbers

The sequences of *K. pneumoniae* RJA166 chromosome (accession number CP019047) and the three plasmids pRJA166a (CP019048), pRJA166b (CP019049) and pRJA166c (CP019050) were deposited in GenBank.

## Supplementary Material

Virulence_MS_RJA166_supplementary_R1_letter_v3.pdf
