# Supplementary material for: Emergence of the third-generation cephalosporin-resistant hypervirulent Klebsiella pneumoniae due to the acquisition of a self-transferable blaDHA-1-carrying plasmid by an ST23 strain
Source: Virulence. 2018 Apr 23;9(1):838–44. doi: 10.1080/21505594.2018.1456229 (PMC5955457; doi:10.1080/21505594.2018.1456229)
Supplement: Virulence_MS_RJA166_supplementary_R1_letter_v3.pdf [file kvir-09-01-1456229-s001.pdf]

Emergence of the third-generation cephalosporin-resistant hypervirulent *Klebsiella pneumoniae* due to the acquisition of a self-transferable *bla*$_{\text{DHA-1}}$-carrying plasmid by an ST23 strain

Yingzhou Xie[1,§], Lijun Tian[2,§], Gang Li[3,§], Hongping Qu[2], Jingyong Sun[4], Wei Liang[5], Xiaobin Li[1], Xiaoli Wang[2], Zixin Deng[1], Jialin Liu[2,*], Hong-Yu Ou[1,6,*]

[1] State Key Laboratory of Microbial Metabolism, Joint International Research Laboratory of Metabolic & Developmental Sciences, School of Life Sciences & Biotechnology, Shanghai Jiao Tong University, Shanghai 200030, China

[2] Department of Critical Care Medicine, Ruijin Hospital, Shanghai Jiao Tong University School of Medicine, Shanghai 200025, China

[3] Department of Laboratory Medicine, Jinshan Hospital Fudan University, Shanghai 201508, China

[4] Department of Clinical Microbiology, Ruijin Hospital, Shanghai Jiao Tong University School of Medicine, Shanghai 200025, China

[5] Department of Laboratory Medicine, the Second People's Hospital of Lianyungang, Jiangsu 222023, China

[6] Shanghai Key Laboratory of Veterinary Biotechnology, Shanghai 200240, China

§These first authors contributed equally to this article.

*To whom correspondence should be addressed: Hong-Yu Ou. Tel: +86 21 62932943; E-mail: hyou@sjtu.edu.cn.

Correspondence may also be addressed to Jialin Liu. Tel: +86 21 53305091; E-mail: ljl11243@rjh.com.cn.

Supplementary data

Supplementary Materials and Methods

Ethics and consent

Strains and primers

String test for the hypermucoviscosity phenotype

PFGE, MLST, and K-typing

Antimicrobial susceptibility test and molecular typing

Killing assay on *Galleria mellonella* larvae

Mouse lethality assay

Antibiotic treatment of the plague in mice

Bacterial load determination

Whole genome sequencing and annotation

Filter mating

Plasmid stability assay

Fitness cost of the acquisition of the bla_{DHA-1}-carrying plasmid pRJA166a

The virulence of transconjugants with pRJA166a is similar to that of recipient

PCR detection of pRJA166a in collected of *K. pneumoniae* clinical isolates

Table S1. Bacterial strains used in this study.

Table S2. Oligonucleotides used in this study.

Table S3. General genome features of the *K. pneumoniae* RJA166, NTUH-K2044, RJF293 and HS11286.

Table S4. Putative virulence factor genes detected on the RJA166 chromosome and the plasmid pRJA166b.

Table S5. Putative acquired antimicrobial resistant genes detected on the RJA166 chromosome and the plasmid pRJA166a.

Figure S1. The comparison of *K. pneumoniae* RJA166 with hvKP NTUH-K2044 and the cKP HS11286.

Figure S2. Histopathology of the liver, spleen, and kidney in the mouse after infection of 10^3 CFU of *K. pneumoniae* RJA166.

Figure S3. The genetic structure of the plasmids pRJA166b and pRJA166c.

Figure S4. The genome-wide single-nucleotide variants (SNVs)-based phylogenetic trees of 107 completely sequenced *K. pneumoniae* chromosomes.

Figure S5. The genetic context of the bla_{DHA-1} gene on the two previously reported plasmids.

Figure S6. The verification of pRJA166a transfer by PFGE and PCR.

Figure S7. The effects of the acquisition of pRJA166a on transconjugants.

Supplementary Materials and Methods

Ethics and consent

The present study is observational, and uses human sputum samples for the *ex vivo* experiments. Sampling from patients is routinely performed in the clinical treatment. For this study, verbal informed consent was obtained from the volunteers. The bacterial samples and data sheets were anonymized. The study protocol, including the procedure for obtaining verbal informed consent, was approved by the ethics committee of the Ruijin Hospital affiliated with Shanghai Jiao Tong University, Shanghai, China. Protocols of mouse experiments were approved by the same ethic committee.

Strains and primers

K. pneumoniae RJA166 was isolated from sputum specimen from a patient in Ruijin Hospital, Shanghai, China. A 47-year-old male patient, with a sign of a cough for over two months and diagnosed with mitral regurgitation, was sent to the cardiac surgery ward on April 18th, 2015. The patient underwent mitral valve repair and coronary artery bypass graft surgery by cardiopulmonary bypass on April 27th, and then, the patient was transferred to the intensive care unit (ICU). However, exploratory thoracotomy was performed because of an unusual volume of bloody drainage on April 28th. Then, the patient coughed up with excessive sputum, and the chest X-ray showed thickness or turbulence of texture in bilateral lungs, with a white blood cell count as high as 19.77×10^9/L on May 3rd. A routine microbiological culture of the sputum sample was performed and *K. pneumoniae* strain RJA166 was isolated on May 4th in the ICU ward. The patient received cefuroxime, vancomycin, and cefotaxime antibiotics during hospitalization, and was discharged from hospital on May 8th.

K. pneumoniae clinical strain RJF293 was collected from a blood sample[1], and its hygromycin-resistant derivative *K. pneumoniae* RJF293H was constructed using a previously described method[2]. The detailed information of strains and primers used in this study are listed in Table S1 and Table S2, respectively.

String test for the hypermucoviscosity phenotype

The string test to determine the hypermucoviscosity phenotype was performed on colonies grown overnight on a blood agar plate at 37°C. The colonies were touched with a loop and pulled upwards, and a string of 5 mm or longer was considered to be a positive result[3].

PFGE, MLST, and K-typing

The pulsed-field gel electrophoresis (PFGE) protocol used was based on the PulseNet 1-day

standardized PFGE protocol for *Escherichia coli* O157:H7 and *Salmonella*[4]. The MLST was performed according to the method described on the BIGSdb website of the Institute Pasteur (http://bigsdb.pasteur.fr/klebsiella/klebsiella.html). Additionally, detection of the capsular serotype of *K. pneumoniae* was conducted using the primer sets listed in a previous publication[5].

Antimicrobial susceptibility test and molecular typing

Bacterial antimicrobial susceptibility was determined using the VITEK 2 Compact system (bioMérieux, Marcy l'Etoile, France). The result was interpreted according to the guidelines of the Clinical and Laboratory Standards Institute guidelines (Twenty-Fourth informational Supplement M10-S26, 2016).

Killing assay on *Galleria mellonella* larvae

The virulence of RJA166 was assessed by a killing assay on *G. mellonella* larvae as per the protocol designed by Insua et al. with minor modification[6]. Larvae weighing 200-400 mg were selected as subjects for infection. Bacteria was cultured overnight in a shaking incubator at 37°C. The bacteria were subcultured by inoculating 30μl overnight culture into 3ml of fresh LB broth (1:100). In a pilot experiment, a culture with an OD600 of 1.0 was determined to contain 10^9 CFU/ml. Thus, when the OD600 of the subculture reached 1.0, 100 μl of the cells were pelleted and resuspended with 900 μl of phosphate-buffered saline (PBS). Ten microlitres of the resuspension, which was equal to 10^6 CFU, was injected into the last right proleg of the larvae using Hamilton precision syringes (Hamilton company). A group was composed of 10 larvae, and each experiment was performed in triplicate. After injection, each group of worms was placed in a 90 mm Petri dish and incubated at 37°C. The mortality was monitored and recorded every 24 hours for a total of 72 hours. The larvae were considered dead when they did not respond to the mechanical stimuli.

Mouse lethality assay

Five-week-old female BALB/c mice were used for virulence assessment, as described previously[7]. Briefly, the overnight cultures of RJA166 to be tested were inoculated into fresh LB broth at 1:100, and grown for 3 hours in a 37°C shaking incubator. Then, the cells were harvested, washed once and re-suspended in saline (0.9%) to an OD600 of 1.0 (approximately 10^9 CFU/ml). Serial dilution was performed to obtain dilutions containing 10^2 CFU/ml, 10^3 CFU/ml, 10^4 CFU/ml and 10^5 CFU/ml for the determination of the median lethal dose (LD50). 100μl of the bacterial suspension was injected

intraperitoneally into the mice. All mice were monitored daily for survival. Five mice were involved in each group for LD50 determination.

Antibiotic treatment of the plague in mice

Thirty-six female BALB/c mice aged 5-6 months were intraperitoneally infected with 1.5×10^3 CFU of *K. pneumoniae* RJA166. The mice then were randomly assigned into 3 groups for different treatment, there were 12 mice in each group. Twelve hours post infection, the mice in the different groups were injected intraperitoneally with ceftazidine (40 mg/kg, q12 h), imipenem (20 mg/kg, q12 h) and 0.9% saline (q12 h). The treatment was stopped when the first death was observed. Two mice in each group were randomly selected for bacterial load determination after the first administration of antibiotics, and the remaining 10 mice were used for survival analysis. The mortality was recorded every 24 hours, for a total of 14 days.

Bacterial load determination

Two mice in each group were sacrificed 12 hours after the first administration of antibiotics. The kidney, spleen and liver of each mouse were sampled and homogenized in 0.9% saline. DNA was extracted from 0.1 g of each homogenate was using the DNeasy Blood & Tissue Kit (Qiagen, catalog No. 69504) according to the user manual. The bacterial load of the tissue was determined by quantitative real-time PCR (qPCR), calculating the relative amount of bacterial gyrase gene *gyrA* to the murine β-actin gene. The gyrase gene and β-actin gene were amplified using the primer sets 166gyrA-qF/R and Bactin-qF/R (Table S2). qPCR was performed using the SYBR Premix Ex Taq II Kit (Takara, catalog No. RR820L) as per the user manual.

Whole genome sequencing and annotation

Since none of the ST23 *K. pneumoniae* strain with multidrug resistance have been completely sequenced to date, we decided to explore the RJA166 antibiotic resistance determinants via whole genome sequencing (WGS). The RJA166 strain was grown overnight at 37°C to stationary phase in LB medium, and the total DNA was isolated from harvested cells. The genome sequence was determined by using Illumina Miseq short-read sequencing (2×251 paired-end reads for a 400-bp library) and PacBio RSII sequencing (4 Kb template). Then, the short and long reads were assembled using SPAdes 3.9 (ref. 8), resulting in four circular replicons (a chromosome and three plasmids). Finally, the genome sequence was annotated using NCBI Prokaryote Genome Annotation Pipeline version 2.0. Additionally, the putative virulence factor, antibiotic resistance determinants, and mobile genetic elements were predicted

using VRprofile[9].

The RJA166 chromosome is 5,348,368 bp in size with a G+C content of 57.6%. It contains 5,354 annotated protein-coding sequences (CDSs). A total of 39 putative virulence factor genes (clusters) were detected on the chromosome (Table S4), including the gene clusters coding for allantoin metabolism (*all*), enterobactin synthesis (*fep*, *ent*), pilus (*yag*), type 3 fimbriae (*mrk*), lipopolysaccharide synthesis (*lpx*) and iron ABC transporter (*kfu*). In addition, twenty-five putative acquired antibiotic resistance determinants are encoded by the RJA166 chromosome (Table S5). The RJA166 chromosome also putatively carries two prophages, two integrative and conjugative elements, and two type VI secretion system gene clusters. Notably, one likely intact 60.3-kb prophage (coordinate 1,411,855 to 1,472,192 bp) is unique to the RJA166 strain and absent in NTUH-K2044, RJF293, HS11286, and other completely sequenced bacterial genomes currently available in GenBank.

The largest plasmid pRJA166a is 230,606 bp in size and its G+C content is 45.8%. It carries four acquired antibiotic resistance genes (Figure 3), namely, $bla_{\text{DHA-1}}$, *qnrB4, sul1,* and $bla_{\text{SHV-12}}$. An intact *tra* gene cluster coding for a type IV secretion system belonging to type F was also predicted on pRJA166a. The plasmid also encodes a relaxase (*RJA_28205*) belonging to the MobH family. The putative *ori*T (coordinate: 141,312-142,312 bp) contains a conserved *nic* motif: 5′- CATCCTG^C-3′ (^: putative *nic*-cleavage site) that has been seen in MobH-associated *ori*T. In addition, a type II toxin-antitoxin locus (TA) was also detected using TAfinder[10], which might aid the stable inheritance of the plasmid by host. This TA locus codes for a RelE-like toxin protein (*RJA_28095*) and a related antitoxin protein containing a PHD domain (*RJA_28090*). These results theoretically confirmed the conjugative ability of pRJA166a [11]. Notably, pRJA166a has a mosaic-like accessory resistance region. For convenience of description and discussion, this accessory resistance region was divided into three putative modules defined on the basis of sequence homology, G+C content, and/or the flanking mobile elements (Figure 6): (i) Module I (3.3 kb, 61.1% G+C) harbours $bla_{\text{SHV-12}}$ and shares 100% full-length nucleotide identity with a region in pKOX_R1, and is flanked by IS*Kpn19* and IS*26*; (ii) Module II (11.5 kb, 51.3% G+C) seemed to cover a conserved $bla_{\text{DHA-1}}$ context and is next to the 3’-CS of the class I integron and IS*CR1* (type I insertion sequences with a common region); and (iii) Module III (1.5 kb, 55.9% G+C) is adjacent to another IS*26* and encodes a glutathione dehydrogenase and an esterase and shares 96% nucleotide identity with pT5282-mphA of *Enterobacter cloacae* T5282 (GenBank accession no: KY270852)[12]. Such a mosaic-like compilation of various functional modules could involve an initial invasion by a mobile

element, containing, for example, a component of module II, which could be followed by recombinational promiscuity in disparate IS elements. This suggests that the accessory resistance region of pRJA166a could be an example of the mechanism by which a bacterial host successfully acquires the peripatetic genetic information of antibiotic resistances from multiple sources.

Like other ST23 hvKP strains, RJA166 also harbours a virulence plasmid, pRJA166b, which is 229 kb in size and has a G+C content of 50.1% (Figure S2a). The mucoviscosity regulator genes *rmpA* and *rmpA2* genes were identified, with no frameshift mutation, on this plasmid. Additionally, the gene clusters coding for the biosynthetic pathway of salmochelin (*iroBCDN*) and aerobactin (*iucABCD*, *shiF*), and for iron uptake regulation (*fecIRA*), were located on this virulence plasmid. No type IV secretion system (T4SS) or gene cluster encoding for other DNA-transfer-associated machinery were predicted to be present on pRJA166b, indicating that the virulence plasmid is non-transferable.

RJA166 carries the third plasmid, pRJA166c, with a size of 111,083 bp and a G+C content of 49.0% (Figure S2b). Homologous plasmids were found in other *K. pneumoniae* strains, including HS11286 and AATZP. By using VRprofile[9], the plasmid pRJA166c was also found to carry a predicted prophage gene cassette (*RJA_30090-RJA_30165*), coding for the prophage proteins such as tail spike and tail fibre protein. This 15-kb region showed homology to the *Salmonella* phage ΦSSU5(ref. 13). These plasmids and the phage ΦSSU5 also showed specific insertion areas, most of which were flanked by transposase/integrase genes (Figure S2c). No gene cluster related to DNA mobilization could be identified on pRJA166c.

Filter mating

Filter mating was performed as described previously[14]. Briefly, overnight cultures of both donor and recipient were inoculated into fresh LB broth at a ratio of 1:100 without any antibiotic. The subcultures were placed in a shaking incubator at 37°C and 220 rpm. When the OD600 of the subcultures reached 1.0, 1 ml of the bacterial cells of both donor and recipient were pelleted and resuspended in 100 μl of 10 mM $MgSO_4$ solution. Donor and recipient cells were mixed at a 1:1 ratio. Then, 20μl of the mixture was dropped on the mixed cellulose ester membrane (pore size 0.45 μm, Merck Millipore, catalog No. HAWG047S6) placed on a pre-warmed LB agar surface. After incubating overnight at 25°C[15], the bacterial cells were washed with 1ml of LB broth and spread on LB agar with the appropriate antibiotics. Dilutions may be used, if necessary. In the present study, *K. pneumoniae* RJA166 was used as the

donor while the three different recipients were *K. pneumoniae* RJF293H, NTUH-K2044IT, and HS11286YZ6 (Table S1), all of which are hygromycin-resistant. Thus, 200 μg/ml hygromycin and 50 μg/ml ceftazidime were added to the LB agar for selection of transconjugants. Experiments were performed in three separate rounds. In addition, each round was repeated in triplicate. The results are presented as (mean±SD).

When transconjugants were observed, three colonies were selected randomly and cultured for verification. PCR with primers (Table S2), unique to the bla_{DHA-1} gene, the backbone of pRJA166a and the chromosomes of RJA166, RJF293H, NTUH-K2044IT and HS11286YZ6, were employed to confirm the transfer of pRJA166a from donor to recipients. *Xba*I-PFGE and S1-nuclease PFGE were also performed for further confirmation of the RJF293H transconjugants.

Plasmid stability assay

The stability of the bla_{DHA-1}-carrying plasmid pRJA166a was tested in both its original host and in the RJF293H transconjugants, as per the previously described protocol[16]. Briefly, 50 lineages for either RJA166 or transconjugant was streaked independently on the LB agar plates without antibiotics. Each passage was approximately equal to 25 generations, as there were approximately 10^8 cells in each colony. The loss of pRJA166a was tested by patching on LB agar containing ceftazidime (50 μg/ml). Three colonies from each passage were randomly selected and verified by PCR amplification of the bla_{DHA-1} gene (*RJA_27775*), the replication protein-encoding gene *repA* (*RJA_27615*) and the type IV secretion apparatus component gene *traD* (*RJA_28210*) on pRJA166a.

Fitness cost of the acquisition of the bla_{DHA-1}-carrying plasmid pRJA166a

It has been reported that the acquisition of resistance plasmids and expression of the AmpC gene could come at an extra fitness cost to bacteria[17,18]. In the head-to-head competition, the ratio of the pRJA166a/RJF293H transconjugant (Tc1) to RJF293H decreased to only 0.02 after 24 hours, with an input ratio at 0.6 (Figure 5a). These results indicated that the acquisition of the large resistance plasmid pRJA166a (231 kb in size) lowered the fitness of the MDR hvKP.

The virulence of transconjugants with pRJA166a is similar to that of recipient

We also compared the growth curve and virulence of pRJA166a-free RJF293H and the pRJA166a/RJF293H transconjugants (Tc1-3) from the filter mating described above. RJF293H and the pRJA166a/RJF293H transconjugants had similar growth rates, which showed that the acquisition of pRJA166a did not affect the primary metabolism too much (Figure 5b). In the killing assay on the greater wax moth larvae, RJF293H and the transconjugants also showed similar virulence (Figure 5c, $p = 0.2695$ by

two-way ANOVA).

PCR detection of pRJA166a in collected of *K. pneumoniae* clinical isolates

We had collected twenty-three hypermucoviscous *K. pneumoniae* isolates, and forty-six cKP isolates in the Ruijin Hospital during the same time period as RJA166 (September 2014 to March 2016). We thus performed PCR to detect the presence of pRJA166a among these isolates using three sets of the primers specific to pRJA166a (Table S2). No isolates, except RJA166, were seen to harbour the $bla_{\text{DHA-1}}$-carrying plasmid pRJA166a.

Table S1 Bacterial strains used in this study

Strain	Genotype*	Key features	Reference
K. pneumoniae			
RJA166	ST23, capsular serotype K1, bla_{DHA-1}	Clinical antibiotic resistance hvKP strain collected from sputum, for example, ceftazidime resistance	This study
NTUH-K2044	ST23, K1 serotype	Clinical hvKP strain collected from liver pus	Wu *et al.*[19]
NTUH-K2044IT	NTUH-K2044::Hm^R	Hygromycin resistant derivative of NTUH-K2044, with the insertion of *hph* gene between *KP1_RS16985* and *KP1_RS16990*	Lab archive
RJF293	ST374, K2 serotype	Clinical hvKP strain collected from blood	Wang *et al.*[3]
RJF293H	RJF293Δ(RJF2_18280-18410)::Hm^R	Hygromycin resistance derivative of RJF293	Lab archive
Tc1, 2 and 3	Transconjugant pRJA166a/RJF293H, bla_{DHA-1}	Hygromycin and ceftazidime resistances	Lab archive
HS11286	ST11, bla_{KPC-2}	Clinical carbapenemase-producing cKP collected from sputum	Liu *et al.*[20]
HS11286YZ6	HS11286Δbla_{KPC-2}Δ(KPHS_p300510-KPHS_p300880)ΔKPHS_44780::Hm^R	Hygromycin resistant derivative of HS11286	Lab archive
E. coli			
HB101	Sm^R	Recipient in conjugation assay	Lab archive
J53	Az^R	Recipient in conjugation assay	Lab archive

* Hm^R, hygromycin resistance; Sm^R, streptomycin resistance; Az^R, sodium azide resistance.

Table S2 Oligonucleotides used in this study.

Primer[a]	Sequence (5'—3')	Purpose
2044SF<br>2044SR	AGACTCCAGCTTGGCGTTAC<br>ACCGCCTTGTTCGATGTGAT	For the PCR detection of DNA fragment (within *KP1_2149*) specific to the *K. pneumoniae* NTUH-K2044 chromosome.
11286SF<br>11286SR	GGGCTAACCAGAAAACCACA<br>GCTTGAGGGGGCTAAAAATC	For the PCR detection of DNA fragment (within *KPHS_44700*) specific to the *K. pneumoniae* HS11286 chromosome.
K1-F<br>K1-R	GGTGCTCTTTACATCATTGC<br>GCAATGGCCATTTGCGTTAG	For the PCR detection of *magA* gene specific to serotype K1 *K. pneumoniae*.
K2-F<br>K2-R	GGATTATGACAGCCTCTCCT<br>CGACTTGGTCCCAACAGTTT	For the PCR detection of *wzy* gene specific to serotype K2 *K. pneumoniae*.
DHA1-F<br>DHA1-R	ATCTGCAACACTGATTTCCG<br>GCACTCAAAATAGCCTGTGC	For the confirmation of $bla_{\text{DHA-1}}$ gene existence.
repA-F<br>repA-R	TTCGAGTCTGGCTGGGAATT<br>CACTCCTCGCTAAAATCCGC	Amplification of the *repA* gene on pRJA166a, to confirm the existence of whole plasmid in transconjugants.
TraD-F<br>TraD-R	GACAAATCAGTCGCCGTGTT<br>GGAAACGTAGGGACAGGGAA	Amplification of the *traD* gene on pRJA166a, to confirm the existence of whole plasmid in transconjugants.
DHA-SF<br>DHA-SR	CGTACGCATACTGGCTTTGC<br>GCAGGATATTCCCGGGATGG	For epidemiologic screening of pRJA166a in the *K. pneumoniae* collection. Specific to the $bla_{\text{DHA-1}}$ gene (*RJA_27775).*
166aJ-SF<br>166aJ-SR	GCTCATCATGGGAAAGCGTT<br>GGCGCTTTATCCGTGTTGAT	For epidemiologic screening of pRJA166a in the *K. pneumoniae* collection. Specific to the junction of IS*26* (*RJA_27695*) and $bla_{\text{SHV-12}}$ gene (*RJA_27685*).
166aI-SF<br>166aI-SR	GCGAACAACGGATAGCACAC<br>GTCGTGTGCAATGACGACAG	For epidemiologic screening of pRJA166a in the *K. pneumoniae* collection. Specific to the *traI* gene (*RJA_28205*).
166gyrA-qF<br>166gyrA-qR	GGTCTCCTTCGGCATCAACA<br>CGCGACGGATCAGTTCGATA	Amplification of the fragment with *K. pneumoniae* RJA166 gyrase gene, used in the quantitative real-time PCR.
Bactin-qF<br>Bactin-qR	TTCCTTCTTGGGTATGGAAT<br>GAGCAATGATCTTGATCTTC	Amplification of the fragment with murine β-actin gene, used in the quantitative real-time PCR.

[a] F indicates the forward primer and R indicates the reverse primer.

Table S3 General genome features of the *K. pneumoniae* RJA166, NTUH-K2044, RJF293 and HS11286[a]

Parameter	RJA166	NTUH-K2044	RJF293	HS11286
hvKP/cKP	hvKP, *bla*$_{DHA-1}$	hvKP	hvKP	cKP, *bla*$_{KPC-2}$
Isolation source	Human, sputum	Human, liver pus	Human, blood	Human, sputum
Capsular serotype	K1	K1	K2	KL103 [b]
MLST	ST23	ST23	ST374	ST11
Chromosome				
size (bp)	5,348,368	5,248,520	5,226,330	5,333,942
No. of annotated CDSs	5,354	5,021	4,995	5,316
No. of putative virulence genes (cluster)	39	39	28	25
No. of putative acquired antibiotic resistance determinants	25	25	25	25
No. of prophages	3	1	1	7
No. of ICEs	2	2	1	2
Plasmid	pRJA166a (resistance)<br>pRJA166b (virulence)<br>pRJA166c	pK2044 (virulence)	pRJF293 (virulence)	pKPHS1 (resistance)<br>pKPHS2 (resistance)<br>pKPHS3 (resistance)<br>pKPHS4<br>pKPHS5<br>pKPHS6
size (bp)	pRJA166a: 230,606<br>pRJA166b: 228,613<br>pRJA166c: 111,083	224,152	224,263	pKPHS1: 122,799<br>pKPHS2: 111,195<br>pKPHS3: 105,974<br>pKPHS4: 3,751<br>pKPHS5: 3,353<br>pKPHS6: 1,308
Incompatibility	pRJA166a: IncHI5<br>pRJA166b: IncHI1B<br>pRJA166c: IncFIB	IncHI1B	IncHI1B	pKPHS1: IncFIB(pKPHS1)<br>pKPHS2: IncR<br>pKPHS3: IncA/C2<br>pKPHS4: Col156<br>pKPHS5: -<br>pKPHS6: Col(KPHS6)
No. of annotated CDSs	pRJA166a: 264<br>pRJA166b: 274<br>pRJA166c: 122	226	229	pKPHS1: 141<br>pKPHS2: 160<br>pKPHS3: 152<br>pKPHS4: 4<br>pKPHS5: 5<br>pKPHS6: 1
No. of putative acquired antibiotic resistance genes	pRJA166a: 5 (including *bla*$_{DHA-1}$)<br>pRJA166b: 0<br>pRJA166c: 0	0	0	pKPHS1: 1 (including *bla*$_{CTX-M-14}$)<br>pKPHS2: 2 (including *bla*$_{KPC-2}$, *bla*$_{TEM-1}$)<br>pKPHS3: 14 (including *bla*$_{CTX-M-14}$, *bla*$_{TEM-1}$, *tetA*, *floR*, *aadA2*, *aacC2*)<br>pKPHS4: 0<br>pKPHS5: 0<br>pKPHS6: 0
No. of putative virulence genes (cluster)	pRJA166a: 0<br>pRJA166b: 3<br>pRJA166c: 0	3	4	None
Reference	This study	Wu, et al.[19]	Wang, et al. [3]	Bi, et al.[2]

[a] hvKP, hypervirulent *K. pneumoniae;* cKP*,* classic *K. pneumonia;* CDS, protein coding sequences.

[b] The capsule serotype of HS11286 was identified by the program Kaptive (https://github.com/katholt/kaptive).

Table S4 Putative virulence factor genes detected on the RJA166 chromosome and the plasmid pRJA166b

Locus tag	Length (aa)	Identities (%)	H_a-value	Hit description
**Chromosome**				
RJA_00810	310	78.2	0.777	*rfaD*, ADP-L-glycero-D-mannoheptose-6-epimerase
RJA_03555	477	70.2	0.686	*rfaE*, ADP-heptose synthase
RJA_04370	302	84.1	0.841	*fimH*, FimH protein precursor
RJA_04375	161	75.2	0.752	*fimG*, FimG protein precursor
RJA_04380	175	70	0.68	*fimF,* FimF protein precursor
RJA_04385	870	77.8	0.767	*fimD,* Outer membrane usher protein fimD precursor
RJA_04390	234	80.8	0.808	*fimC*, Chaperone protein fimC precursor
RJA_04395	178	73.1	0.702	*fimI,* Fimbrin-like protein fimI precursor
RJA_04400	182	81.3	0.813	*fimA*, Type-1 fimbrial protein, A chain precursor
RJA_04410	202	82.8	0.787	*fimE*, Type 1 fimbriae Regulatory protein fimE
RJA_04415	209	83.5	0.799	*fimB,* Type 1 fimbriae Regulatory protein fimB
RJA_04450	202	100	1	*mrkA,* type 3 fimbriae major subunit
RJA_04455	241	100	0.967	*fimB,* Type 1 fimbriae Regulatory protein fimB
RJA_04460	828	100	1	*mrkC*, type 3 fimbriae usher protein
RJA_04465	331	100	1	*mrkD*, type 3 fimbriae adhesin
RJA_04470	211	100	1	*mrkF,* type 3 fimbriae anchor protein
RJA_04475	238	93.2	0.929	*mrkJ,* Regulator of Type III fimbriae
RJA_04480	190	99.5	0.995	*mrkI,* Transcriptional regulator
RJA_04485	234	100	1	*mrkH,* c-di-GMP-Dependent Transcriptional Activator
RJA_05395	343	65.5	0.647	*chuS,* heme oxygenase ChuS
RJA_05770	171	73.1	0.731	*luxS*, S-ribosylhomocysteinase [AI-2 (VF0406)]
RJA_06410	191	64.9	0.649	*algU*, alginate biosynthesis protein AlgZ/FimS
RJA_08870	374	79.2	0.786	*gmd,* GDP-mannose 4,6-dehydratase [O-antigen (VF0392)]
RJA_08875	322	74	0.733	*fcl*, GDP-fucose synthetase [O-antigen (VF0392)]
RJA_08905	456	73.5	0.735	*manB*, phosphomannomutase [O-antigen (VF0392)]
RJA_09380	210	89.5	0.895	*rmpA*, regulator of mucoid phenotype
RJA_09405	409	98.8	0.988	*iroD*, salmochelin siderophore ferric enterochelin esterase
RJA_09410	1214	98	0.98	*iroC*, salmochelin siderophore ATP-binding cassette
RJA_09415	371	97.3	0.973	*iroB*, salmochelin siderophore glycosyltransferase
RJA_09430	726	93	0.927	*iroN*, salmochelin receptor
RJA_09505	673	99.7	0.997	*fyuA*, pesticin/yersiniabactin receptor protein
RJA_09510	525	99.8	0.998	*ybtE*, yersiniabactin siderophore biosynthetic protein
RJA_09515	245	99.2	0.992	*ybtT*, yersiniabactin biosynthetic protein
RJA_09520	366	99.7	0.997	*ybtU*, yersiniabactin biosynthetic protein
RJA_09525	3163	100	1	*irp1*, yersiniabactin biosynthetic protein
RJA_09530	2035	99.9	0.999	*irp2*, yersiniabactin biosynthetic protein
RJA_09535	319	100	1	*ybtA*, transcriptional regulator YbtA
RJA_09540	570	99.8	0.998	*ybtP*, lipoprotein inner membrane ABC-transporter
RJA_09545	600	100	1	*ybtQ*, inner membrane ABC-transporter YbtQ

RJA_09550	426	99.5	0.995	*ybtX*, putative signal transducer
RJA_09555	434	99.8	0.998	*ybtS*, salicylate synthase Irp9
RJA_10725	284	83	0.827	*kdsA*, 2-dehydro-3-deoxyphosphooctonate aldolase
RJA_10900	300	74.4	0.717	*galU*, glucosephosphate uridylyltransferase
RJA_12075	193	66	0.653	*sodB*, superoxide dismutase
RJA_13015	226	70.9	0.699	*mgtC*, Mg2+ transport protein
RJA_15305	163	95.7	0.957	*hcp*, Hcp family type VI secretion system effector
RJA_16920	729	98.5	0.985	*iutA*, ferric aerobactin receptor precusor
RJA_17510	342	100	1	*kfuC*, Fe(3+) ions import ATP-binding protein
RJA_17515	524	100	1	*kfuC*, Fe(3+) ions import ATP-binding protein
RJA_17520	338	100	1	*kfuA*, iron ABC transporter substrate-binding protein
RJA_17625	364	85.4	0.835	*ompA*, outer membrane protein A
RJA_17825	248	66.5	0.673	*kdsB*, 3-deoxy-manno-octulosonate cytidylyltransferase
RJA_17845	582	66.4	0.66	*msbA*, lipid transporter ATP-binding/permease
RJA_19630	251	89.1	0.88	*entA*, 2,3-dihydro-2,3-dihydroxybenzoate dehydrogenase
RJA_19635	283	86.7	0.873	*entB*, isochorismatase
RJA_19640	535	81.1	0.802	*entE*, 2,3-dihydroxybenzoate-AMP ligase component of enterobactin synthase multienzyme complex
RJA_19645	391	75.2	0.752	*entC*, isochorismate synthase 1
RJA_19650	319	84.6	0.793	*fepB*, ferrienterobactin ABC transporter periplasmic binding protein
RJA_19655	413	84.5	0.847	*entS*, enterobactin exporter, iron-regulated
RJA_19660	335	86.2	0.857	*fepD*, ferrienterobactin ABC transporter permease
RJA_19665	330	83	0.83	*fepG*, iron-enterobactin ABC transporter permease
RJA_19670	264	88.6	0.883	*fepC*, ferrienterobactin ABC transporter ATPase
RJA_19675	1293	78.2	0.78	*entF*, enterobactin synthase multienzyme complex component, ATP-dependent
RJA_19685	402	66.4	0.659	*fes*, enterobactin/ferric enterobactin esterase
RJA_19695	742	81.5	0.825	*fepA*, ferrienterobactin outer membrane transporter
RJA_20490	297	100	1	*arcC*, carbamate kinase
RJA_20495	267	100	1	*ylbF*, anaerobic allantoin catabolic oxamate carbamoyltransferase
RJA_20500	419	100	1	*ylbE*, putative cytoplasmic protein
RJA_20505	555	100	1	*fdrA*, NAD(P)-binding acyl-CoA synthetase
RJA_20510	349	100	1	*allD*, ureidoglycolate dehydrogenase
RJA_20515	411	100	1	*allC*, allantoate amidohydrolase
RJA_20520	260	100	1	*KP1_1371*, putative glyoxylate utilization gene
RJA_20525	381	100	1	*glxK*, glycerate kinase
RJA_20530	436	100	1	*ybbY*, purine permease
RJA_20535	453	100	1	allB, allantoinase
RJA_20540	484	100	1	*ybbW*, allantoin permease
RJA_20545	418	100	1	*KP1_1364*, probable metabolite transport protein
RJA_20550	292	100	1	*glxR*, tartronic semialdehyde reductase
RJA_20555	258	100	1	*hyi*, hydroxypyruvate isomerase
RJA_20560	593	99.8	0.998	*gcl*, glyoxylate carboligase

RJA_20565	272	100	1	*allR*, regulator of the allantoin metabolism gene cluster
RJA_20570	160	100	1	*allA*, ureidoglycolate hydrolase
RJA_20580	307	100	1	*allS*, transcriptional activator of the allantoin metabolism gene cluster
RJA_21545	221	87.7	0.869	*yagV*/*ecpE*, E. coli common pilus chaperone EcpE
RJA_21550	547	94.1	0.941	*yagW*/*ecpD*, polymerized tip adhesin of ECP fibers
RJA_21555	841	93.1	0.931	*yagX*/*ecpC*, E. coli common pilus usher EcpC
RJA_21560	222	91.4	0.914	*yagY*/*ecpB*, E. coli common pilus chaperone EcpB
RJA_21565	195	95.4	0.954	*yagZ*/*ecpA*, E. coli common pilus structural subunit EcpA
RJA_21570	180	90.6	0.906	*ykgK*/*ecpR*, regulator protein EcpR
RJA_21875	193	79.2	0.788	*gmhA*/*lpcA*, phosphoheptose isomerase
RJA_22000	271	69	0.69	*IlpA*, immunogenic lipoprotein A
RJA_22085	262	67.6	0.676	*lpxA*, UDP-N-acetylglucosamine acyltransferase
RJA_22095	341	65.1	0.645	*lpxD*, UDP-3-O-(3-hydroxymyristoyl) glucosamine N-acyltransferase
RJA_22295	140	68.1	0.657	*gspG*, general secretion pathway protein G
RJA_22600	305	77.6	0.774	*lpxC*, UDP-3-O-(R-3-hydroxymyristoyl)-N-acetylglucosamine deacetylase
RJA_25085	548	75.8	0.724	*htpB*, Hsp60, 60K heat shock protein HtpB
RJA_25575	752	72.1	0.694	*fepA*, ferrienterobactin outer membrane transporter
**Plasmid**				
RJA_28710	1214	98	0.98	*iroC*, ATP binding cassette transporter
RJA_28715	371	99.2	0.992	*iroB*, glucosyltransferase IroB
RJA_29895	733	89.6	0.895	*iutA*, ferric aerobactin receptor precusor IutA
RJA_29900	425	100	1	*iucD*, L-lysine 6-monooxygenase IucD
RJA_29905	577	100	1	*iucC*, aerobactin siderophore biosynthesis protein IucC
RJA_29910	315	100	1	*iroB*, Salmochelin siderophore glycosyltransferase
RJA_29915	574	100	1	*iucA*, aerobactin siderophore biosynthesis protein
RJA_30045	210	94.3	0.943	*rmpA*, regulator of mucoid phenotype
RJA_30070	726	97.9	0.977	*iroN*, salmochelin receptor IroN
RJA_30075	409	99	0.99	*iroD,* Salmochelin siderophore ferric enterochelin esterase

[a] Detection the putative virulence factors with the protein sequence similarities by using BLASTp-based H_a-value
To examine the degree of sequence similarities at an amino acid level between each query protein and the VRprofile-collected virulence factors[9], the NCBI BLASTp-derived H_a-value was employed. For each query, the H_a-value was calculated as follows:

$$H_{\mathrm{a}} = i \times \frac{lm}{lq}$$

where *i* was the level of BLASTp identities of the region with the highest Bit score expressed as a frequency of between 0 and 1, l_m the length of the highest scoring matching sequence (including gaps) and l_q the query length. If there were no matching sequences with a BLASTp E value < 0.01, the H_a-value assigned to that query sequence was defined as zero[21]. Therefore H_a-value belonged to the set, $H_a \in [0,1]$. Here, a strict H_a-value cut-off ≥ 0.64 was used to determine the significant sequence similarities; for example, the identities is 80% and the ratio of matching length is 80%.

Table S5 Putative acquired antimicrobial resistant genes detected on the RJA166 chromosome and the plasmid pRJA166a[a]

Gene	Coordinate	Locus_tag	Description
Chromosome			
emrD	29520..30704	*RJA_00150*	Multidrug resistance efflux pump
arnA	297416..299401	*RJA_01455*	Resistance to polymyxin
acrB	460091..463201	*RJA_02365*	Multidrug resistance efflux pump
acrA	463214..464353	*RJA_02370*	Multidrug resistance efflux pump
bacA	676621..677442	*RJA_03530*	Resistance to bacitracin
tolC	690855..692324	*RJA_03600*	Multidrug resistance efflux pump
acrB	1341288..1344401	*RJA_06885*	Multidrug resistance efflux pump
bcr	1591430..1592626	*RJA_08260*	Bcr drug resistance efflux transporter
mdtK	2336736..2338109	*RJA_12040*	Multidrug resistance efflux pump
PBP2	2528391..2530256	*RJA_13045*	Penicillin-binding protein 2
$bla_{\text{SHV-12}}$	2742881..2743741	*RJA_14190*	Resistance to beta-lactam
mdtH	3302719..3303927	*RJA_17090*	Multidrug resistance efflux pump
mdtG	3310430..3311657	*RJA_17150*	Multidrug resistance efflux pump
macB	3494128..3496068	*RJA_18010*	Multidrug resistance efflux pump
mdtM	3534297..3535529	*RJA_18215*	Multidrug resistance efflux pump
rosA	4022868..4024088	*RJA_20670*	Resistance to fosmidomycin, efflux pump
rosB	4024314..4025990	*RJA_20685*	Resistance to fosmidomycin, efflux pump
acrA	4041480..4042673	*RJA_20760*	Multidrug resistance efflux pump
acrB	4042696..4045842	*RJA_20765*	Multidrug resistance efflux pump
tet34	4245595..4246053	*RJA_21800*	Resistance to tetracycline
ampC	4396482..4397336	*RJA_22530*	Resistance to beta-lactam
ksgA	4475680..4476501	*RJA_22895*	Resistance to kasugamycin
mdtM	4605201..4606442	*RJA_23610*	Multidrug resistance efflux pump
fosA	4613564..4613983	*RJA_23660*	Resistance to fosfomycin
mdtL	5342074..5343252	*RJA_27355*	Multidrug resistance efflux pump
pRJA166a [b]			
$bla_{\text{SHV-12}}$	57916..58776	*RJA_27685*	Resistance to beta-lactam
qnrB4	65549..66196	*RJA_27735*	Resistance to quinolone
$bla_{\text{DHA-1}}$	70317..71456	*RJA_27775*	Resistance to beta-lactam
sul1	73033..73872	*RJA_27795*	Resistance to sulphonamide

[a] The antimicrobial resistant genes were predicted by VRprofile (http://bioinfo-mml.sjtu.edu.cn/VRprofile/)[9].

[b] pRJA166a has a frameshift in the region (189,027..189,664), resulting in the pseudogene of the mucoid phenotype regulator gene *rmpA2*.

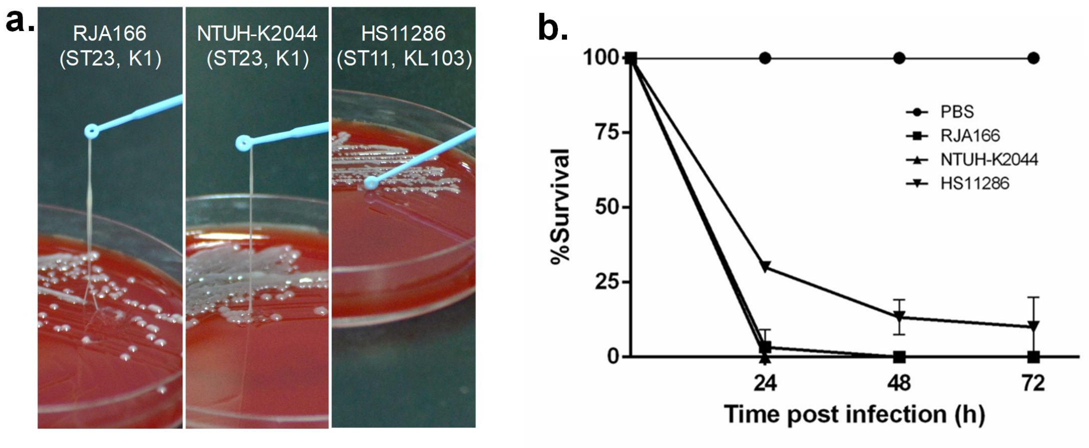

Figure S1. The comparison of *K. pneumoniae* RJA166 with hvKP NTUH-K2044 and the cKP HS11286. (a) Positive hypermucoviscosity phenotype of *K. pneumoniae* RJA166 evidenced by the string test. After the overnight incubation at 37°C on blood agar, a mucoviscous string over 40 mm in length was observed when lifting the RJA166 colony using a disposable inoculation loop. The K1 and ST23 hvKP strain NTUH-K2044 was used for positive control while the ST11 cKP strain HS11286 was used as a negative control. (b) Killing assays on *Galleria mellonella* larvae infected with *K. pneumoniae* RJA166. Within 24 hours after injection with a dosage of 10^6 CFU, all the larvae were killed in the NTUH-K2044 and RJA166 groups, while a survival rate of 15% was seen in the HS11286 group.

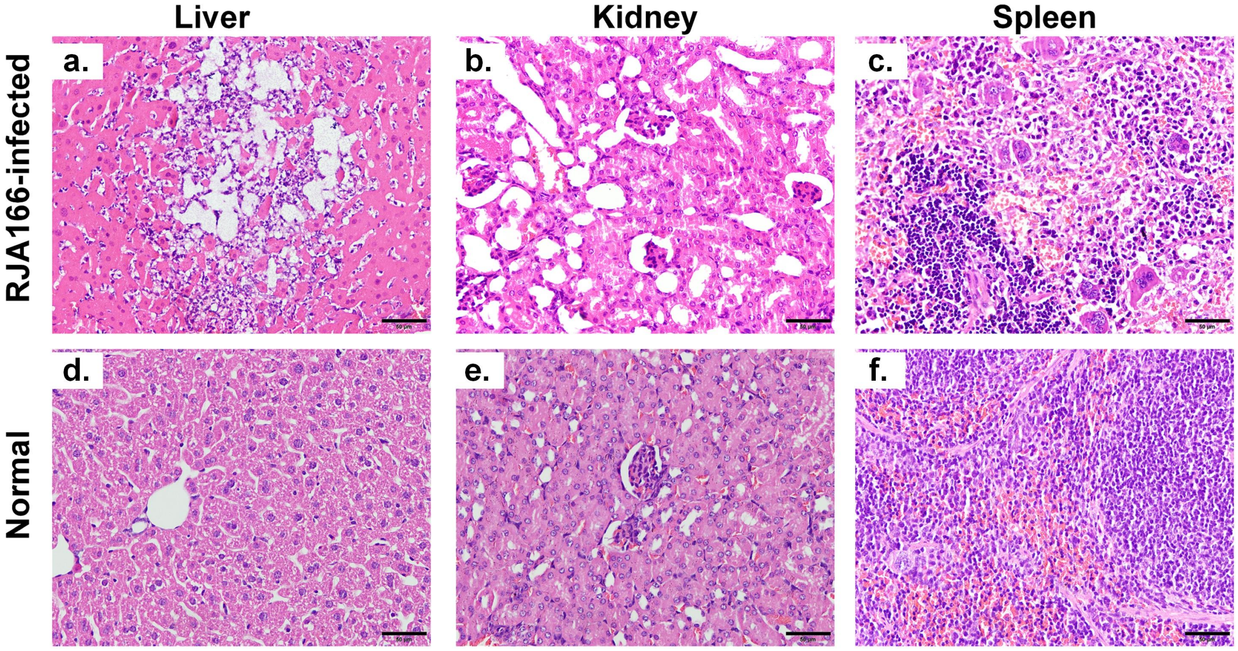

Figure S2. Histopathology of the liver, spleen, and kidney in the mouse after infection of 10^3 CFU of *K. pneumoniae* RJA166. Tissues were taken 3 days after the mouse challenged with bacteria and stained by hematoxylin and eosin. (a) to (c) show the tissues from the RJA166-infected mouse, while (d) to (f) are those for the mouse injected with 0.9% saline as control.

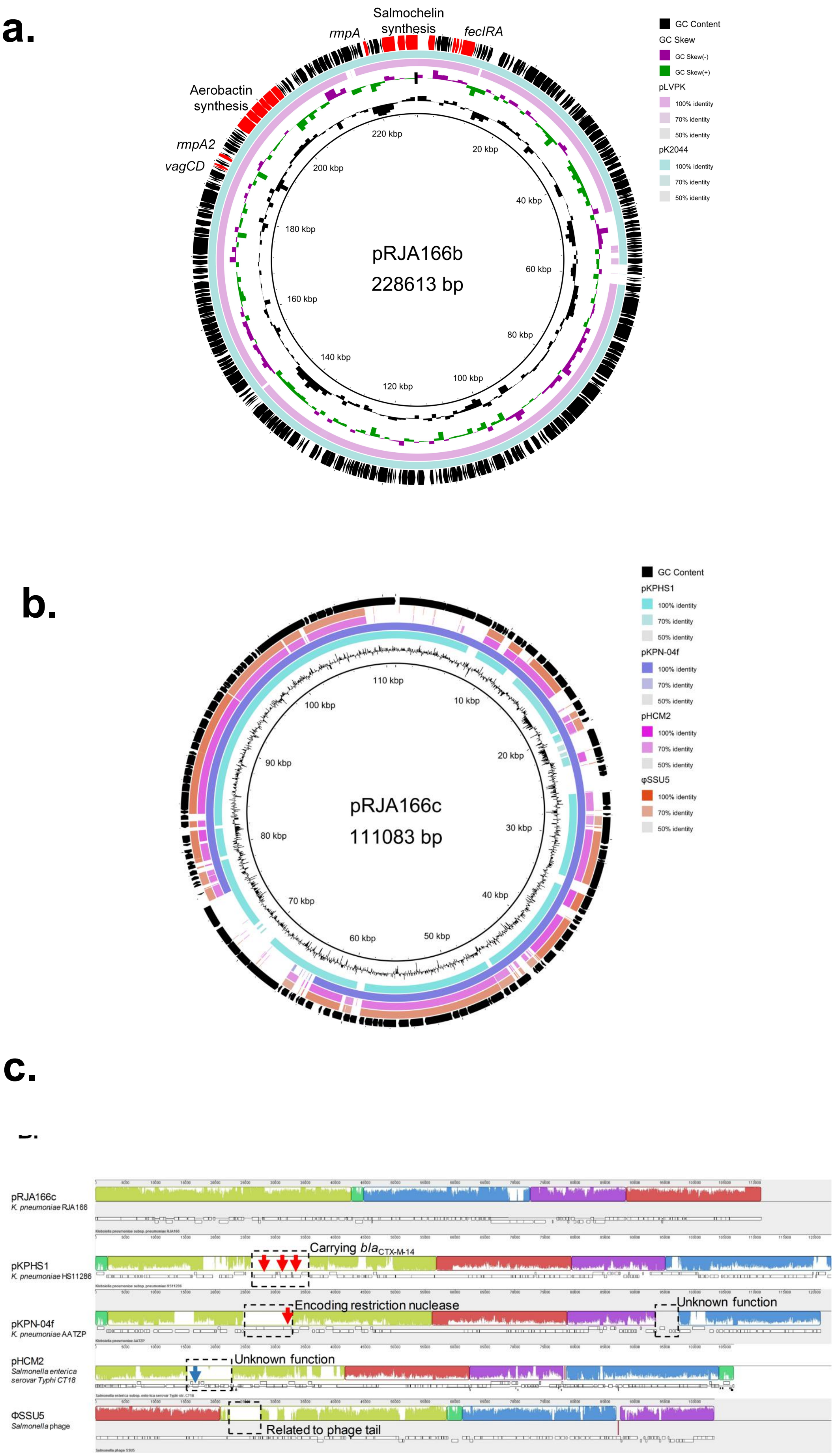

a.
Salmochelin synthesis
rmpA
fecIRA
Aerobactin synthesis
rmpA2
vagCD
220 kbp
20 kbp
200 kbp
40 kbp
180 kbp
60 kbp
160 kbp
80 kbp
140 kbp
120 kbp
100 kbp
pRJA166b
228613 bp
GC Content
GC Skew
GC Skew(-)
GC Skew(+)
pLVPK
100% identity
70% identity
50% identity
pK2044
100% identity
70% identity
50% identity
b.
110 kbp
10 kbp
100 kbp
20 kbp
90 kbp
30 kbp
80 kbp
40 kbp
70 kbp
60 kbp
50 kbp
pRJA166c
111083 bp
GC Content
pKPHS1
100% identity
70% identity
50% identity
pKPN-04f
100% identity
70% identity
50% identity
pHCM2
100% identity
70% identity
50% identity
φSSU5
100% identity
70% identity
50% identity
c.
pRJA166c
K. pneumoniae RJA166
pKPHS1
K. pneumoniae HS11286
Carrying $bla_{CTX-M-14}$
pKPN-04f
K. pneumoniae AATZP
Encoding restriction nuclease
Unknown function
pHCM2
Salmonella enterica serovar Typhi CT18
Unknown function
ΦSSU5
Salmonella phage
Related to phage tail

Figure S3. The genetic structure of the plasmids pRJA166b, pRJA166c. (a) The plasmid pRJA166b was aligned with well-documented virulence *K. pneumoniae* plasmids pK2044 (GenBank accession: NC_006625) and pLVPK (NC_005249). The outernost ring shows the identified protein-coding genes of pRJA166b, where the gene/gene cluster encoding for virulence factors are shown in red. (b) pRJA166c was aligned to three homologous plasmids, namely, pKPHS1 (Genbank accession: CP003223)[20], pKPN-04f (CP014756) and pHCM2 (NC_003385)[22], and to the *Salmonella* phage ΦSSU5 (NC_018843)[13]. Predicted CDSs are shown on the outermost ring. (c) Alignment generated by Mauve[23]. Specific insertion regions are boxed in the dashed lines, and the biological significance is shown on the side. Genes encoding transposase and integrase are indicated by red and blue downward arrows, respectively.

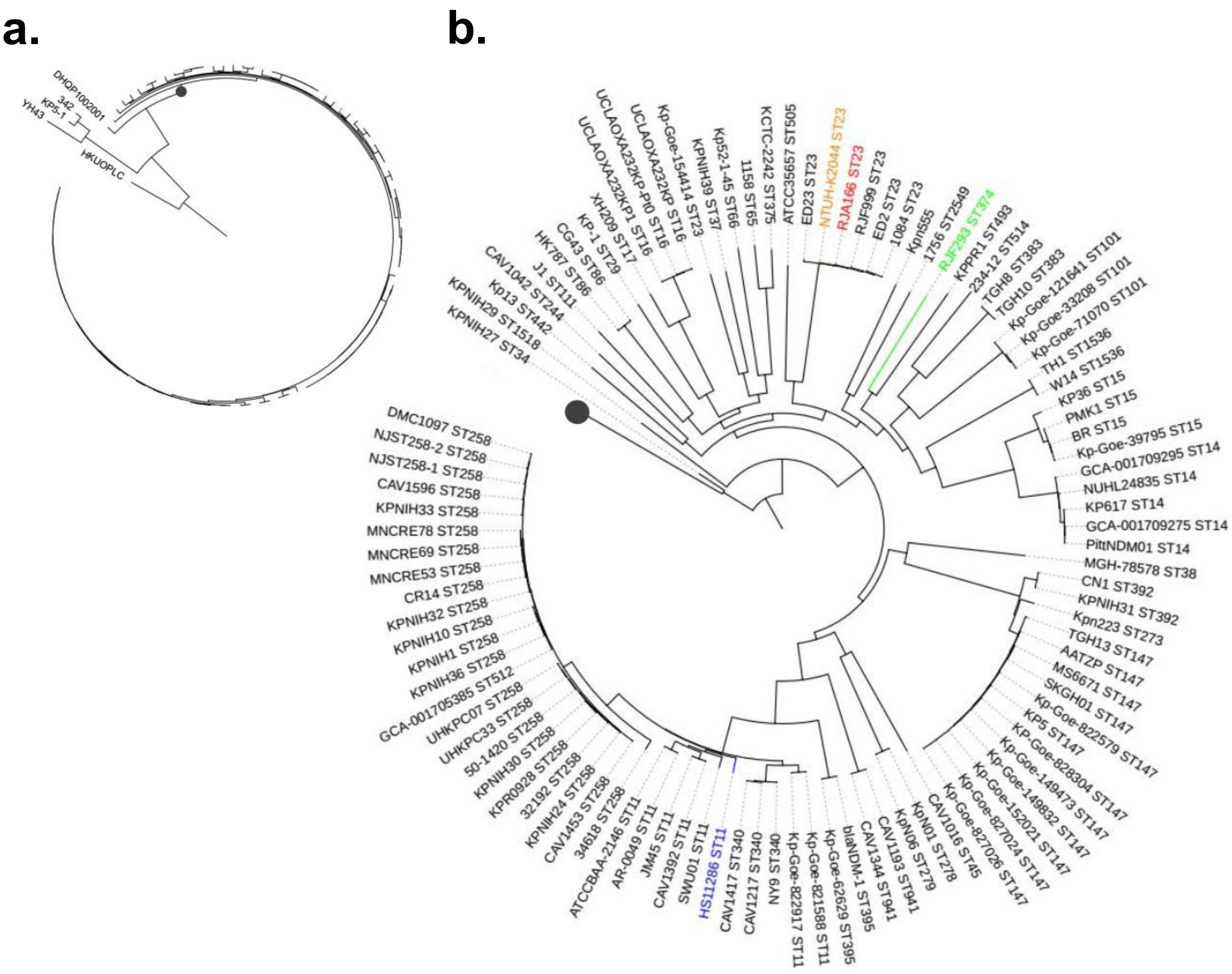

Figure S4. The genome-wide single-nucleotide variants (SNVs)-based phylogenetic trees of 107 completely sequenced *K. pneumoniae* chromosomes. (a) The phylogeny scheme generated from 33,784 core SNVs for all genomes using kSNP3 with the *k*=21 (ref. 24) and displayed by iTOL with midpoint rooting[25]. (b) SNV tree as in panel (a) but after removal of five nodes (HKUOPLC, YH43, KP5-1, 342, DHQP1002001). Four *K. pneumoniae* isolates listed in the Supplementary Table S2 are highlighted by colour (red, RJA166; orange, NTUH-K2044; green, RJF293; blue, HS11286).

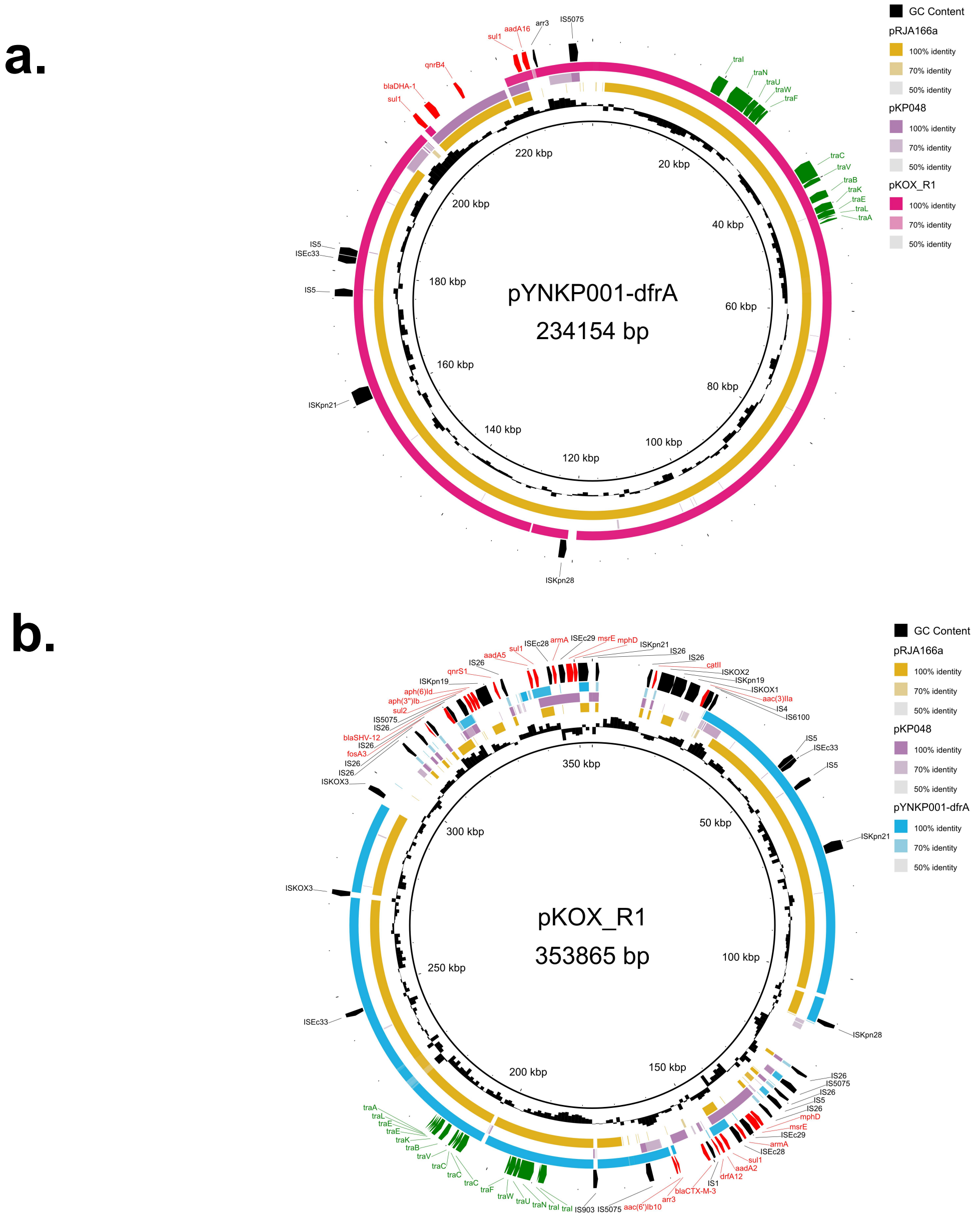

Figure S5. The genetic context of the *bla*$_{DHA-1}$ gene on the two previously reported plasmids.

(a) The genetic context of the *bla*$_{DHA-1}$ gene on pRJA166a with comparison with the two previously reported plasmids pYNKP001-dfrA of *Raoultella ornithinolytica* YNKP001[5] and (b) pKOX_R1 of *Klebsiella michiganensis* E718[6]. The predicted antibiotic resistance gene is shown in red, the *tra* gene cluster is in green, and the IS element is shown in black.

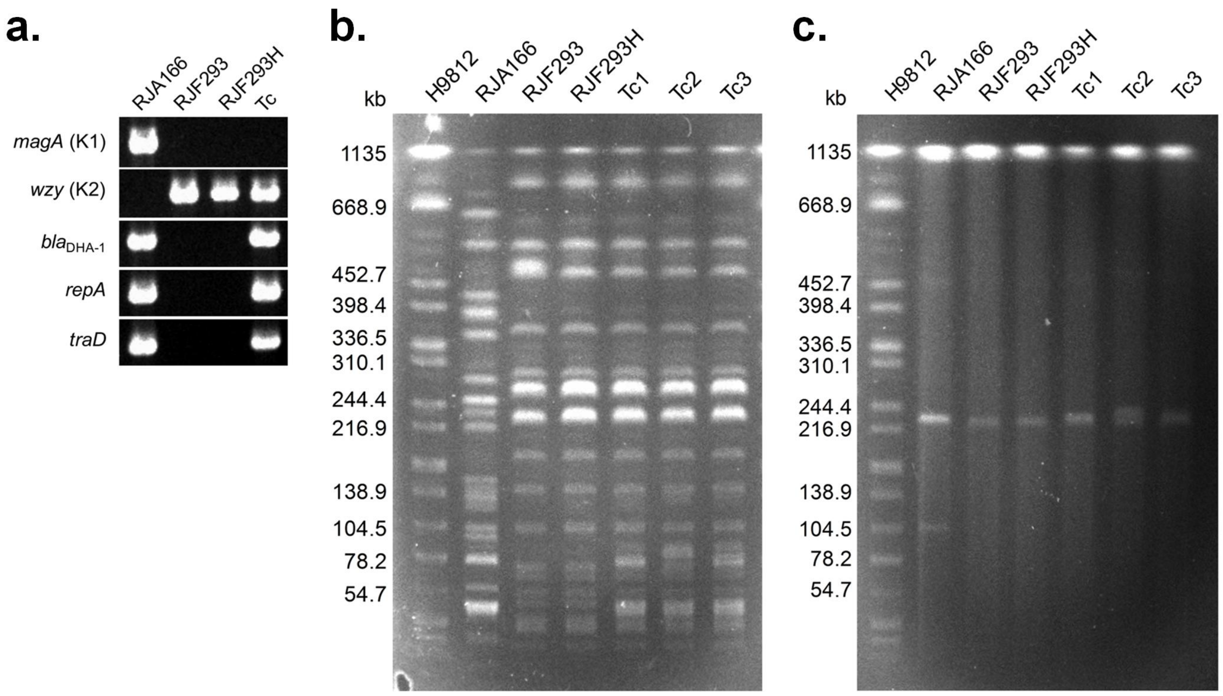

Figure S6. The verification of pRJA166a transfer by PFGE and PCR. (a) The transfer of pRJA166a was tested by PCR. The successful amplifications of *bla*$_{DHA-1}$, *traD* and *repA* genes revealed the transfer of the entire pRJA166a plasmid to the recipient in a conjugation assay. (b) Three randomly selected pRJA166a transconjugants (Tc1, 2 and 3) showed the same *Xba*I-PFGE pattern as the recipient RJF293H and different pattern from the donor RJA166. (c) The pRJA166a/RJF293H transconjugants were verified using S1-nuclease treatment followed by PFGE. All the transconjugants and the recipient RJF293H showed one band on the gel (two plasmids with indistinguishable sizes), while the donor RJA166 showed two bands.

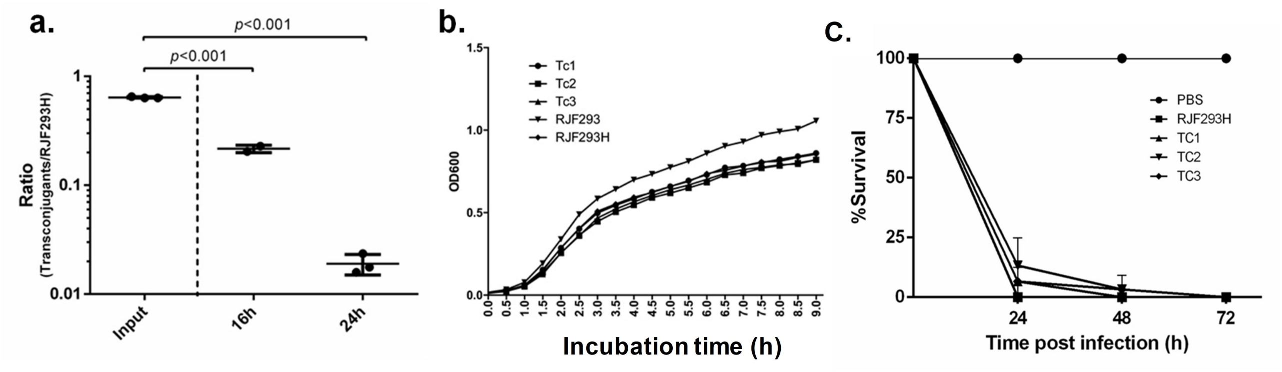

Figure S7. The effects of the acquisition of pRJA166a on transconjugants. (a) The pRJA166a/RJF293H transconjugant-to-RJF293H ratio in the competition assay. The result of the competition assay is presented as the ratio of transconjugant (Tc1) to RJF293H. CFU was counted and the ratio was calculated at 16 h and 24 h after the mixing of these two strains. RJF293 outcompeted the transconjugant (Tc1) significantly at the two time points (both exhibited $p<0.001$ by Student's t test); (b)Growth curves of *K. pneumoniae* RJF293, RJF293H and transconjugants. Three transconjugants (Tc 1-3) were randomly selected and tested. Bacteria were cultured in LB broth at 37°C. All the transconjugants and RJF293H showed similar growth rates, which were lower than the wild-type RJF293 strain. (c)Killing assays on *Galleria mellonella*. Larvae were injected with PBS or with 10^6 CFU of *K. pneumoniae* RJF293H (recipient in the conjugation assay of pRJA166a), and three pRJA166a/RJF293H transconjugants (Tc1, 2 and 3). No significant difference could be detected between them.

REFERENCES

1. Wang X, Xie Y, Li G, Liu J, Li X, Tian L, Sun J, Ou H-Y, Qu H. Whole-Genome-Sequencing characterization of bloodstream infection-causing hypervirulent *Klebsiella pneumoniae* of capsular serotype K2 and ST374. Virulence [Internet] 2018; 5594:00–00. Available from: https://www.tandfonline.com/doi/full/10.1080/21505594.2017.1421894
2. Bi D, Jiang X, Sheng ZK, Ngmenterebo D, Tai C, Wang M, Deng Z, Rajakumar K, Ou HY. Mapping the resistance-associated mobilome of a carbapenem-resistant *Klebsiella pneumoniae* strain reveals insights into factors shaping these regions and facilitates generation of a "resistance-disarmed" model organism. J Antimicrob Chemother 2015; 70:2770–4.
3. Shon AS, Bajwa RPS, Russo TA. Hypervirulent (hypermucoviscous) *Klebsiella pneumoniae*. Virulence [Internet] 2013; 4:107–18. Available from: http://www.tandfonline.com/doi/abs/10.4161/viru.22718
4. Ribot EM, Fair MAA, Gautom R, Cameron DNN, Hunter SBB, Swaminathan B, Barrett TJ. Standardization of pulsed-field gel electrophoresis protocols for the subtyping of *Escherichia coli* O157:H7, *Salmonella*, and *Shigella* for PulseNet. Foodborne Pathog Dis [Internet] 2006; 3:59–67. Available from: http://online.liebertpub.com/doi/abs/10.1089/fpd.2006.3.59?url_ver=Z39.88-2003&rfr_id=ori%3Arid%3Acrossref.org&rfr_dat=cr_pub%3Dpubmed&
5. Chen Z, Liu M, Cui Y, Wang L, Zhang Y, Qiu J, Yang R, Liu C, Zhou D. A novel PCR-based genotyping scheme for clinical *Klebsiella pneumoniae*. Future Microbiol [Internet] 2014; 9:21–32. Available from: http://han.bg.umed.lodz.pl/han/wok/www.futuremedicine.com/doi/abs/10.2217/fmb.13.137
6. Insua JL, Llobet E, Moranta D, Pérez-Gutiérrez C, Tomás A, Garmendia J, Bengoechea JA. Modeling *Klebsiella pneumoniae* pathogenesis by infection of the wax moth *Galleria mellonella*. Infect Immun 2013; 81:3552–65.
7. Arvanitis M, Li G, Li DD, Cotnoir D, Ganley-Leal L, Carney DW, Sello JK, Mylonakis E. A conformationally constrained cyclic acyldepsipeptide is highly effective in mice infected with methicillin-susceptible and -resistant *Staphylococcus aureus*. PLoS One 2016; 11:1–10.
8. Bankevich A, Nurk S, Antipov D, Gurevich AA, Dvorkin M, Kulikov AS, Lesin VM, Nikolenko SI, Pham S, Prjibelski AD, et al. SPAdes: A new genome assembly algorithm and its applications to single-cell sequencing. J Comput Biol [Internet] 2012; 19:455–77. Available from: http://online.liebertpub.com/doi/abs/10.1089/cmb.2012.0021
9. Li J, Tai C, Deng Z, Zhong W, He Y, Ou H-Y. VRprofile: gene-cluster-detection-based profiling of virulence and antibiotic resistance traits encoded within genome sequences of pathogenic bacteria. Brief Bioinform [Internet] 2017; :bbw141. Available from: https://academic.oup.com/bib/article-lookup/doi/10.1093/bib/bbw141
10. Shao Y, Harrison EM, Bi D, Tai C, He X, Ou HY, Rajakumar K, Deng Z. TADB: A web-based resource for Type 2 toxin-antitoxin loci in bacteria and archaea. Nucleic Acids Res 2011; 39:606–11.
11. Bi D, Liu L, Tai C, Deng Z, Rajakumar K, Ou H-YY. SecReT4: a web-based bacterial type IV secretion system resource. Nucleic Acids Res [Internet] 2013; 41:D660-5. Available from: http://www.ncbi.nlm.nih.gov/pubmed/23193298
12. Liang Q, Yin Z, Zhao Y, Liang L, Feng J, Zhan Z, Wang H, Song Y, Tong Y, Wu W, et al. Sequencing and comparative genomics analysis of the IncHI2 plasmids pT5282-mphA and p112298-catA and the IncHI5 plasmid pYNKP001-dfrA. Int J Antimicrob Agents [Internet] 2017; 49:709–18. Available from: http://linkinghub.elsevier.com/retrieve/pii/S0924857917301085
13. Kim M, Kim S, Ryu S. Complete genome sequence of bacteriophage SSU5 specific for *Salmonella enterica* serovar Typhimurium rough strains. J Virol [Internet] 2012; 86:10894. Available from:

http://www.pubmedcentral.nih.gov/articlerender.fcgi?artid=3457314&tool=pmcentrez&rendertype=abstract

14. Lesic B, Carniel E. Horizontal transfer of the high-pathogenicity Island of *Yersinia pseudotuberculosis*. J Bacteriol 2005; 187:3352–8.

15. Sherburne CK, Lawley TD, Gilmour MW, Blattner FR, Burland V, Grotbeck E, Rose DJ, Taylor DE. The complete DNA sequence and analysis of R27, a large IncHI plasmid from *Salmonella typhi* that is temperature sensitive for transfer. Nucleic Acids Res [Internet] 2000; 28:2177–86. Available from: http://www.pubmedcentral.nih.gov/articlerender.fcgi?artid=105367&tool=pmcentrez&rendertype=abstract

16. Sun J, Yang R-S, Zhang Q, Feng Y, Fang L-X, Xia J, Li L, Lv X-Y, Duan J-H, Liao X-P, et al. Co-transfer of *bla*$_{NDM-5}$ and *mcr-1* by an IncX3-X4 hybrid plasmid in *Escherichia coli*. Nat Microbiol [Internet] 2016; 1:16176. Available from: http://www.ncbi.nlm.nih.gov/pubmed/27668643

17. Chiara Di Luca M, Sørum V, Starikova I, Kloos J, Hü lter N, Naseer U, Johnsen PJ, Samuelsen Ø. Low biological cost of carbapenemase-encoding plasmids following transfer from *Klebsiella pneumoniae* to *Escherichia coli*. J Antimicrob Chemother 2016; :10–4.

18. Pérez-Gallego M, Torrens G, Castillo-Vera J, Moya B, Zamorano L, Cabot G, Hultenby K, Albertí S, Mellroth P, Henriques-Normark B, et al. Impact of AmpC derepression on fitness and virulence: the mechanism or the pathway? MBio [Internet] 2016; 7:1–12. Available from: http://www.ncbi.nlm.nih.gov/pubmed/27795406

19. Wu KM, Li NH, Yan JJ, Tsao N, Liao TL, Tsai HC, Fung CP, Chen HJ, Liu YM, Wang JT, et al. Genome sequencing and comparative analysis of *Klebsiella pneumoniae* NTUH-K2044, a strain causing liver abscess and meningitis. J Bacteriol 2009; 191:4492–501.

20. Liu P, Li P, Jiang X, Bi D, Xie Y, Tai C, Deng Z, Rajakumar K, Ou HY. Complete genome sequence of *Klebsiella pneumoniae subsp. pneumoniae* HS11286, a multidrug-resistant strain isolated from human sputum. J Bacteriol 2012; 194:1841–2.

21. Shao Y, He X, Harrison EM, Tai C, Ou H-YY, Rajakumar K, Deng Z. mGenomeSubtractor: a web-based tool for parallel in silico subtractive hybridization analysis of multiple bacterial genomes. Nucleic Acids Res [Internet] 2010; 38:W194-200. Available from: http://www.ncbi.nlm.nih.gov/pubmed/20435682

22. Kidgell C, Pickard D, Wain J, James K, Diem Nga LT, Diep TS, Levine MM, O'Gaora P, Prentice MB, Parkhill J, et al. Characterisation and distribution of a cryptic *Salmonella typhi* plasmid pHCM2. Plasmid [Internet] 2002; 47:159–71. Available from: http://www.ncbi.nlm.nih.gov/pubmed/12151231

23. Darling ACE, Mau B, Blattner FR, Perna NT. Mauve: multiple alignment of conserved genomic sequence with rearrangements. Genome Res [Internet] 2004; 14:1394–403. Available from: http://www.ncbi.nlm.nih.gov/pubmed/15231754

24. Gardner SN, Slezak T, Hall BG. kSNP3.0: SNP detection and phylogenetic analysis of genomes without genome alignment or reference genome. Bioinformatics 2015; 31:2877–8.

25. Letunic I, Bork P. Interactive tree of life (iTOL) v3: an online tool for the display and annotation of phylogenetic and other trees. Nucleic Acids Res [Internet] 2016; 44:W242–5. Available from: https://www.ncbi.nlm.nih.gov/pmc/articles/PMC4987883/
